# Influence of different positions on hemodynamics derived from noninvasive transcutaneous Doppler ultrasound

**DOI:** 10.1002/phy2.62

**Published:** 2013-09-20

**Authors:** Cangel Pui-yee Chan, Pui-ling Cheung, Mandy Man Tse, Nandini Agarwal, Sangeeta Narain, Stewart Siu-Wa Chan, Brendan E Smith, Colin A Graham, Timothy H Rainer

**Affiliations:** 1 Accident and Emergency Medicine Academic Unit, Prince of Wales Hospital, The Chinese University of Hong KongNew Territories, Hong Kong; 2 School of Biomedical Science, Charles Sturt UniversityBathurst, NSW, Australia; 3 Intensive Care Unit, Bathurst Base HospitalBathurst, NSW, Australia

**Keywords:** Doppler ultrasonography, hemodynamics, patient positioning

## Abstract

A proper alignment of the ultrasound beam to the aortic or pulmonary outflow tracts is essential to acquire accurate signals. This study aimed to investigate the influence of different positions on the acquisition of Doppler signals using a noninvasive transcutaneous Doppler ultrasound. This was a prospective observational crossover study. Two operators performed hemodynamics measurements on each subject in supine, sitting, semirecumbent, passive leg raising (PLR) 20°, and PLR 60° positions using both aortic and pulmonary approaches. All Doppler flow profile images were assessed using the Fremantle and Prince of Wales Hospital criteria. Time required to obtain Doppler signals was recorded. A total of 60 subjects (50% males) aged 18–60 years old were investigated. In both sitting and semirecumbent positions, aortic stroke volume indexes (SVIs) and cardiac indexes (CIs) were significantly lower than those in the other three positions while the pulmonary CIs were comparable to that in the supine position. In the sitting position, the aortic signal qualities were lower and the time to obtain the pulmonary Doppler signals was prolonged. Instead, the signal quality and the time to obtain the Doppler signals in the semirecumbent position were similar to those in the other three positions using the pulmonary approach. PLR did not cause a significant increase in SVI regardless of the degree of leg elevation. These data show that it is feasible to perform the noninvasive transcutaneous Doppler ultrasound using the pulmonary approach in the semirecumbent position for patients unable to maintain the supine position. The aortic approach in the sitting and semirecumbent positions is not suitable as it is not sufficiently reliable.

## Introduction

Early recognition of hemodynamic instability is important in emergency care. The Ultrasonic Cardiac Output Monitor (USCOM) is capable of measuring hemodynamic parameters noninvasively and appears to be simple and rapid to use, portable, relatively inexpensive and has less potential complications compared with the standard technique, pulmonary artery catheterization (Tan et al. 2005; Wong et al. 2008; Phillips et al. 2009; Thom et al. 2009; Duchateau et al. 2011). It provides accurate and reliable data to monitor hemodynamic status (Nguyen et al. 2006; van Lelyveld-Haas et al. 2008; Su et al. 2008; Cattermole et al. 2010; Chan et al. 2012). USCOM is now being recognized as a standard of care for fluid management and has been included in the guidelines within the Intraoperative Fluid Management Technologies Pack by the United Kingdom NHS since May 2012 (NHS, 2012).

Like other ultrasonic devices, USCOM measurement is operator dependent. It is critical to have proper alignment of the ultrasound beam to the aortic or pulmonary outflow tracts in order to acquire accurate signals. However, no guidelines on patient position are available to facilitate optimal measurement of hemodynamic parameters using USCOM. Siu et al. (2008) reported the impact of five different patient positions including supine, Trendelenburg, left lateral tilt, right lateral tilt, and sitting on USCOM measurement. However, the semirecumbent position, which is a more clinically preferred position in critically ill patients with concurrent increased intracranial pressure or respiratory compromise, was not investigated. On the other hand, the Trendelenburg position caused only a slight increase in cardiac preload volume and did not significantly improve cardiac performance (Reuter et al. 2003). Also, use of the Trendelenburg position was associated with adverse consequences and it was recommended to avoid (Bridges and Jarquin-Valdivia 2005; Kalmar et al. 2010; Molloy 2011). In contrast, passive leg raising (PLR) is a useful clinical guide to fluid resuscitation and can be used for effective autotransfusion (Monnet and Teboul 2008; Axelsson et al. 2010). Most importantly, Siu et al. (2008) did not compare the difference in values of hemodynamic parameters among different patient positions and did not use pulmonary approach to measure hemodynamic parameters.

This study aims (i) to determine the difference in stroke volume index (SVI) for sitting and semirecumbent positions compared to supine position, the standard position for USCOM measurement, on aortic and pulmonary measurements using USCOM, (ii) to evaluate the effect of different positions on the quality of Doppler signals, (iii) to determine the differences in time required to obtain Doppler signals between different positions, and (iv) to explore the differences in SVI between positions with and without volume loading.

## Methods

### Study design

This was a prospective observational crossover study conducted at the Emergency Department (ED) of the Prince of Wales Hospital (PWH) in Hong Kong to investigate the effect of different patient positions on the measurement of hemodynamic parameters using the USCOM. The study was approved by the Clinical Research Ethics Committee of our university. Written consent was obtained from all recruited volunteers after they received a detailed explanation about the purpose of the study.

### Inclusion and exclusion criteria

Chinese adult volunteers aged 18–60 years old were recruited for the measurement of hemodynamic parameters using the USCOM. Volunteers who were below 18 years of age, unable to give consent, pregnant, lactating, known cardiac disease, or previous cardiac surgery were excluded.

### Sample size estimation

According to the results of our pilot study (*n* = 8), the mean aortic SVI in the supine, sitting, and semirecumbent positions were 38.9 ± 6.6 mL m^−2^, 22.1 ± 7.3 mL m^−2^, and 28.5 ± 7.8 mL m^−2^, respectively. Therefore, a minimum sample size of eight subjects was required per group to achieve a power of 80% with a two-sided significance level of α = 0.05. In order to investigate the difference in SVI between the three positions, and the two aortic and pulmonary windows, the minimum sample size required for this study is 48 subjects (8 × 3 positions × 2 valves = 48). An extra 25% of subjects were recruited in case for unforeseen circumstances. The minimum sample size was therefore set at 60.

### USCOM measurements and data collection

Two operators received hands-on USCOM training sessions and performed aortic and pulmonary measurements on 50 healthy volunteers. The intra- and interoperator coefficients of variation were <10% and the intraclass correlation coefficient was over 0.87 compared to the skill of a senior researcher with extensive experience on the use of USCOM acting as a “gold standard”.

Following the completion of the training, the operators were allowed to recruit volunteers for the USCOM measurement. The USCOM allows beat-to-beat measurement of hemodynamic parameters by measuring blood flow across the heart valves using continuous wave Doppler ultrasound and FlowTracer's automatic signal tracking. An acoustic image can be obtained from either the aortic or pulmonary outflow tracts.

The USCOM calculated SV by measuring the ejection velocity of blood flow across the aortic or pulmonary valves and multiplying this by the cross-sectional area of the outflow tract diameter which was based on the volunteer's weight and height. The USCOM also measured HR and therefore gave a calculated CO (CO = SV × HR). SVI and CI are SV and CO indexed to body surface area.

**Figure 1:**
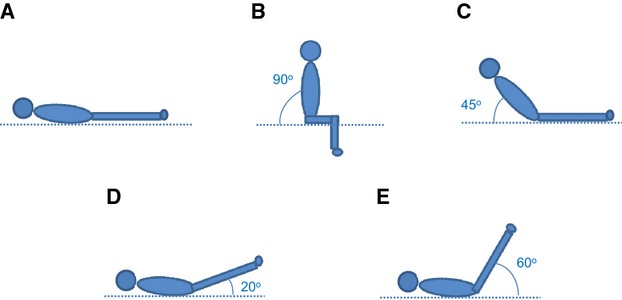
Five different positions: (A) supine, (B) sitting, (C) semirecumbent, (D) PLR 20°, and (E) PLR 60°

**Figure 2:**
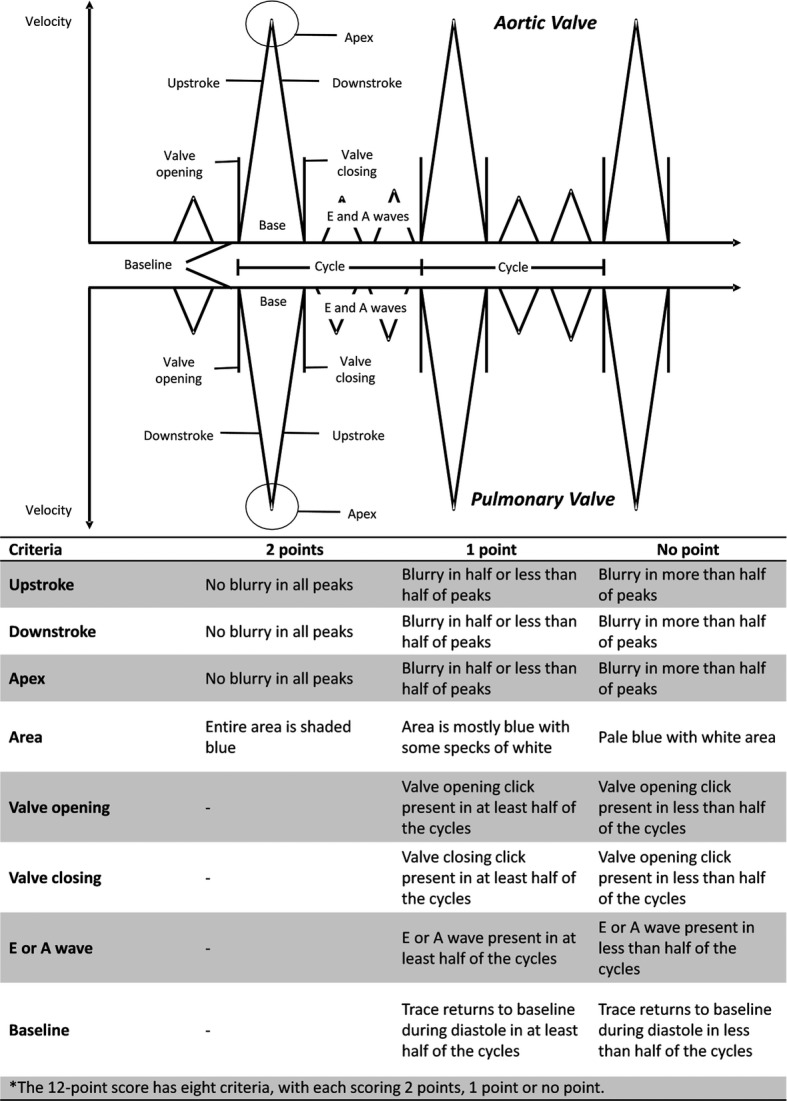
Prince of Wales Hospital (PWH) Criteria for assessment of quality of Doppler signal.

Using both aortic and pulmonary approaches, USCOM measurements were performed in the supine, sitting, semirecumbent, PLR 20°, and PLR 60° positions (Fig. 1). Blood pressure was measured before the USCOM measurement in each position. For the semirecumbent position, the head of bed was raised to 45°. For the PLR position, the legs of a volunteer in a supine position were raised to 20° and 60°, respectively. A protractor was used to measure the degree of the angle. A computer-generated random number was used to randomly allocate the sequence of positions for USCOM measurement for each volunteer to minimize bias from progressive ease of measurements on the same volunteer due to practice. Each position was maintained for 5 min before the BP and USCOM measurements. When two PLR positions were allocated consecutively, the volunteer returned to the supine position for 2 min after the first PLR position. For the USCOM measurement, a transducer was placed on the chest in either the suprasternal position to measure transaortic blood flow in each position or the left parasternal position to measure transpulmonary blood flow. At least three consecutive cycles were required for each scan. Each volunteer was scanned three times per operator. All Doppler flow profile images were recorded in the USCOM device and then assessed by two senior researchers independently first using the Fremantle criteria (Dey and Sprivulis 2005) and second using the PWH criteria (Fig. 2). They were blinded to the position assigned in the USCOM measurement at the time of image review. Both the Fremantle and PWH criteria were used for assessing the quality of the Doppler signals. The Fremantle criteria developed by Dey and Sprivulis is a 6-point scoring system with six criteria and each scoring one point or no point (Dey and Sprivulis 2005). The PWH criteria developed by our research team is a 12-point scoring system with eight criteria and each scoring two points, one point or no point (Cattermole et al. 2009). The time required to obtain three Doppler signals with the highest SVIs was recorded by an independent researcher.

Demographics of all participants including sex, age, height, weight, body mass index (BMI), HR, systolic blood pressure (SBP), diastolic blood pressure (DBP), and respiration rate (RR) were obtained. Blood pressure was measured with an appropriately sized cuff using a standard oscillometric device in the ED (Mindray VS-800 Vital Sign Monitor; Mindray Co. Ltd., Shenzhen, China).

### Outcome measures

The primary outcome was the aortic and pulmonary SVIs for sitting and semirecumbent positions compared to supine position using USCOM. The secondary outcomes were the Fremantle and PWH scores, the time required to obtain three Doppler signals using aortic and pulmonary approaches among different positions, and also the SVIs between positions with and without volume loading.

### Statistical analysis

All data were presented as median, interquartile range, mean ± standard deviation as appropriate. Continuous variables were analyzed using paired Student *t*-tests, Wilcoxon test, and one-way ANOVA tests followed by post hoc Bonferroni tests and least significant difference (LSD) tests as appropriate. Interoperator variability for SVI measurement and interrater variability for assessment of signal quality based on the Fremantle and PWH criteria were determined using (i) intraclass correlation, which used the one-way random effects model for absolute agreement of single paired observations with operators or raters selected at random, (ii) Bland–Altman limits of agreement which were calculated for the percentage difference in SVIs obtained by the two operators or in scores given by the two raters, and (iii) coefficients of variation which were calculated as the percentage ratios of the SDs and the means of the SVIs obtained by the two operators or the scores given by the two raters. Descriptive statistics and data comparison were carried out using PASW Statistics v18.0.0 (SPSS Inc., IBM Corporation, Chicago, IL) and MedCalc v11.5.1 (MedCalc Software, Mariakerke, Belgium). A *P* value of <0.05 was considered as statistically significant.

## Results

A total of 60 volunteers (50% male) aged 38.5 years (IQR: 25) were recruited in this study from 18 October 2011 to 27 February 2012. The subjects were 163.5 ± 8.0 cm in height and 58.7 ± 8.3 kg in weight. They had a BMI of 21.9 ± 1.9 and respiratory rate of 15 ± 3 per minute. There was no significant difference in SBP among different positions (114 ± 21 to 122 ± 16 mmHg). Subjects in the sitting (77 ± 10 mmHg) and semirecumbent (74 ± 14 mmHg) positions had significantly higher DBP than in the other three positions (67 ± 13 to 70 ± 10 mmHg) (*P* < 0.05) although were clinically irrelevant.

### Hemodynamics in different positions

**Figure 3:**
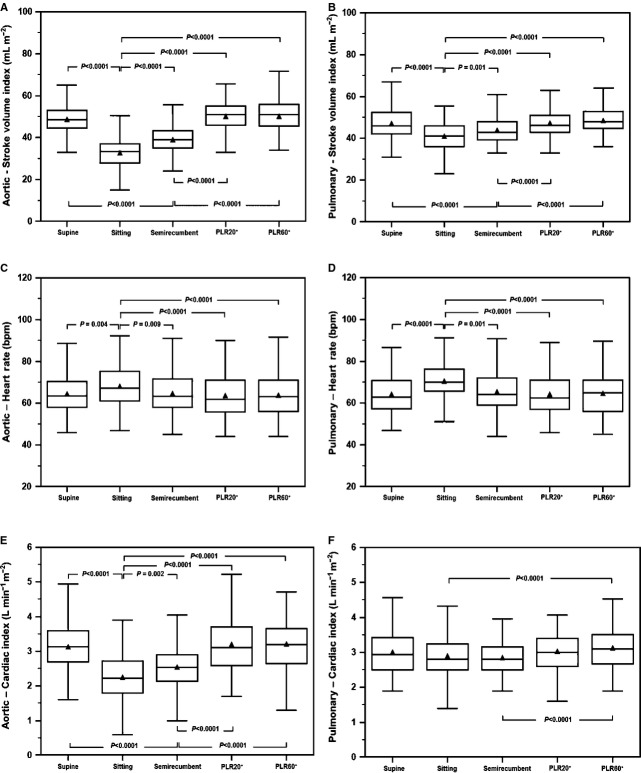
Box plots showing the stroke volume indexes (A and B), heart rates (C and D), and cardiac indexes (E and F) in 60 volunteers using aortic and pulmonary approaches among five different positions. The difference across different positions were significant (ANOVA test, *P* < 0.0001) while differences between positions are shown above the relevant boxes in the graph (Bonferroni test). The lines inside the boxes denoted the medians while the boxes marked the interval between the 25th and 75th percentiles. The whiskers denoted the interval between the 10th and 90th percentiles, and the means were illustrated as triangles.

Using both aortic and pulmonary approaches, the SVI in the sitting position (aortic 32.8 ± 9.0 mL m^−2^, pulmonary 41.2 ± 6.7 mL m^−2^) was significantly lower than those in the supine (aortic 48.7 ± 7.7 mL m^−2^, pulmonary 47.1 ± 7.0 mL m^−2^), semirecumbent (aortic 39.1 ±8.6 mL m^−2^, pulmonary 43.9 ± 6.1 mL m^−2^), PLR 20° (aortic 50.2 ± 8.4 mL m^−2^, pulmonary 47.5 ± 6.4 mL m^−2^), and PLR 60° positions (aortic 50.3 ± 8.3 mL m^−2^, pulmonary 48.6 ± 6.8 mL m^−2^; Fig. 3A and B). The SVI in the semirecumbent position was also significantly lower than those in the supine, PLR 20° and PLR 60° positions. Using the post hoc LSD, the SVI in the PLR 60° position was significantly higher than that in the supine position for the pulmonary measurement (*P* = 0.026).

The heart rate in the sitting position (aortic 68 ± 10 bpm, pulmonary 71 ± 9 bpm) was significantly higher than those in the supine (aortic 65 ± 10 bpm, pulmonary 64 ± 10 bpm), semirecumbent (aortic 65 ± 10 bpm, pulmonary 66 ± 9 bpm), PLR 20° (aortic 64 ± 10 bpm, pulmonary 64 ± 10 bpm), and PLR 60° positions (aortic 64 ± 10 bpm, pulmonary 65 ± 10 bpm) for both aortic and pulmonary measurements (Fig. 3C and D).

Using the aortic approach, the CI in the sitting position (aortic 2.3 ± 0.7 L min^−1^ m^−2^, pulmonary 2.9 ± 0.6 L min^−1^ m^−2^) was significantly lower than those in the supine (aortic 3.1 ± 0.6 L min^−1^ m^−2^, pulmonary 3.0 ± 0.6 L min^−1^ m^−2^), semirecumbent (aortic 2.5 ± 0.7 L min^−1^ m^−2^, pulmonary 2.9 ± 0.5 L min^−1^ m^−2^), PLR 20° (aortic 3.2 ± 0.8 L min^−1^ m^−2^, pulmonary 3.0 ± 0.5 L min^−1^ m^−2^), and PLR 60° positions (aortic 3.2 ± 0.8 L min^−1^ m^−2^, pulmonary 3.1 ± 0.6 L min^−1^ m^−2^; Fig. 3E). The CI in the semirecumbent position was also significantly lower than those in the supine, PLR 20°, and PLR 60° positions. For the pulmonary measurements, the CI in the PLR 60° position was significantly higher than those in the sitting and semirecumbent positions (Fig. 3F).

**Figure 4:**
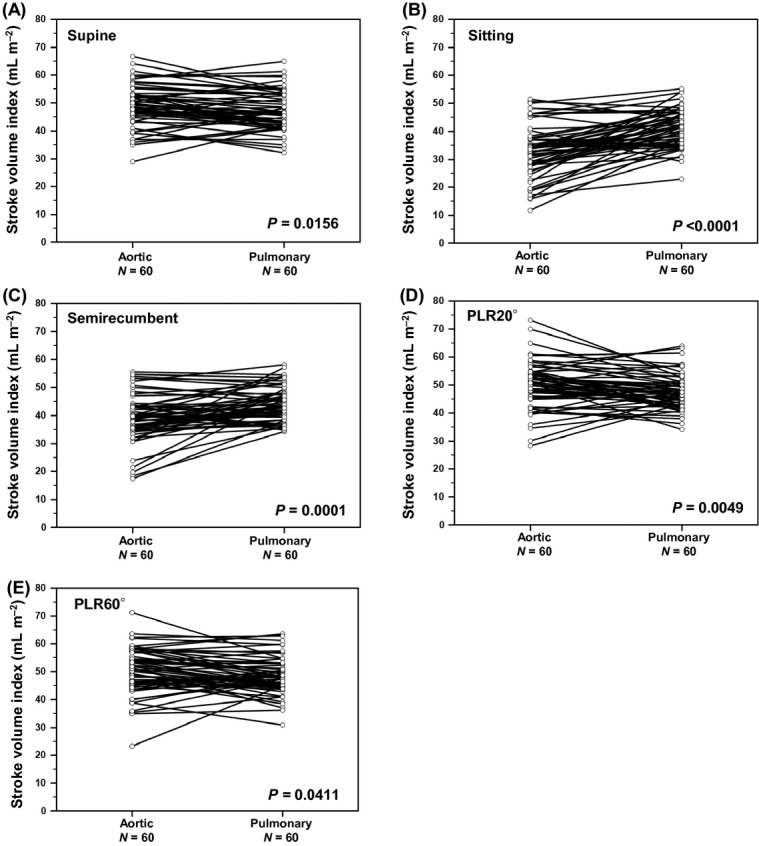
Comparison of the stroke volume indexes (SVIs) using aortic and pulmonary approaches in the (A) supine, (B) sitting, (C) semirecumbent, (D) passive leg raising (PLR) 20°, and (E) PLR 60° positions using Wilcoxon test.

The SVIs using aortic approach were significantly higher than those using the pulmonary approach in the supine, PLR 20°, and PLR 60° positions but opposite findings were observed in the sitting and semirecumbent positions (Fig. 4).

A total of 33 (55%) subjects were scanned independently by a second operator. The interoperator variability for SVI measurement determined by coefficient of variation, intraclass correlation, and Bland–Altman limits of agreement is shown in Table 1. The interoperator reliability and agreement were good.

**Table 1:** Interoperator variation for the measurement of SVI

Parameters	Stroke volume index
Number of subjects	33
Coefficient of variation among different positions (%)	5.1–8.8
Intraclass correlation (95% CI)	0.94 (0.93 to 0.95)
Bland-Altman limits of agreement (%)	
Upper limit (95% CI)	22.3 (20.2 to 24.4)
Lower limit (95% CI)	−22.3 (−24.4 to −20.2)

### Quality of Doppler signal in different positions

The interrater variability for assessment of signal quality based on the Fremantle and PWH criteria was determined by coefficient of variation, intraclass correlation, and Bland–Altman limits of agreement (Table 2). The interrater reliability and agreement were good for both Fremantle and PWH criteria.

**Table 2:** Interrater variation for the Fremantle criteria and the PWH criteria

Parameters	Fremantle criteria	PWH criteria
Number of subjects	60	60
Coefficient of variation (%)	11.2	8.9
Intraclass correlation (95% CI)	0.93 (0.92 to 0.94)	0.89 (0.87 to 0.91)
Bland-Altman limits of agreement (%)		
Upper limit (95% CI)	11.9 (11.0 to 12.7)	9.4 (8.6 to 10.2)
Lower limit (95% CI)	−12.4 (−13.3 to −11.6)	−13.4 (−14.2 to −12.6)

The PWH scores significantly correlated with the Fremantle scores (*r* = 0.6424, *P* < 0.0001). There were also significant correlations between the PWH scores and the SVIs (*r* = 0.6166, *P* < 0.0001), and between the Fremantle scores and the SVIs (*r* = 0.3574, *P* < 0.0001).

#### Fremantle criteria

Using the aortic approach, the Fremantle score for the sitting position was significantly lower than those for the supine, PLR 20°, PLR 60° (*P* < 0.0001), and semirecumbent (*P* = 0.014) positions (Table 3). Besides, the Fremantle score for the semirecumbent position was significantly lower than those for the PLR 20° (*P* = 0.039) and PLR 60° (*P* = 0.007) positions. Nevertheless, the Fremantle scores for the pulmonary measurements were similar across all positions (*P* > 0.05) and were significantly higher than those for the aortic measurements among all positions (*P* < 0.0001).

**Table 3:** Scores of Doppler signals based on Fremantle and PWH criteria in five different positions

	Fremantle criteria			PWH criteria		
		
	Aortic	Pulmonary	*P*^5^	Aortic	Pulmonary	*P*^5^
Supine	4.0 (1.0)	5.0 (0.0)	<0.0001	10.0 (1.0)	10.0 (1.0)	0.623
Sitting	4.0 (0.0)^1^	5.0 (0.0)	<0.0001	9.0 (1.0)^2^	10.0 (2.0)^3^	0.0002
Semirecumbent	4.0 (1.0)^4^	5.0 (0.0)	<0.0001	10.0 (1.0)	10.0 (1.0)	0.120
PLR20°	4.0 (1.0)	5.0 (0.0)	<0.0001	10.0 (1.0)	10.0 (1.0)	0.244
PLR60°	4.0 (1.0)	5.0 (0.0)	<0.0001	10.0 (1.0)	10.0 (1.0)	0.938

Data presented as median (IQR). ANOVA test followed by post hoc Bonferroni test across five positions.

PLR, passive leg raising; PWH, Prince of Wales Hospital.

1 Sitting compared with supine, PLR20°, PLR60° (*P* < 0.0001), and semirecumbent (*P* = 0.014).

2 Semirecumbent compared with PLR20° (*P* = 0.039) and PLR60° (*P* = 0.007).

3 Sitting compared with supine, semirecumbent, PLR20°, and PLR60° (*P* < 0.0001).

4 Sitting compared with supine (*P* = 0.012) and PLR60° (*P* = 0.001).

5 Wilcoxon test.

#### PWH criteria

Using the aortic approach, the PWH score for the sitting position was significantly lower than those for the supine, semirecumbent, PLR 20°, and PLR 60° positions (*P* < 0.0001; Table 3). The PWH score for the sitting position was also significantly lower than those for the supine (*P* = 0.012) and PLR 60° (*P* = 0.001) positions using the pulmonary approach. Besides, the PWH scores for the pulmonary measurements were significantly higher than those for the aortic measurements in the sitting position (*P* = 0.0002).

### Time for three Doppler signals in different positions

As shown in Table 4, the time required to obtain three Doppler signals with the highest SVIs using the aortic approach in the semirecumbent position was significantly longer than that in the supine position (*P* = 0.007). Using the pulmonary approach, the time required in the sitting position was significantly longer than that in PLR 20° position (*P* = 0.037). In addition, the time required to obtain three Doppler signals using the aortic approach was significantly shorter than that using the pulmonary approach in all positions (*P* < 0.05).

**Table 4:** Time required to obtain three Doppler signals among five different positions

	Aortic	Pulmonary	*P*^3^
Supine	135s ± 51s	214s ± 116s	<0.0001
Sitting	167s ± 61s	253s ± 161s^2^	0.0002
Semirecumbent	174s ± 57s^1^	212s ± 105s	0.016
PLR20°	146s ± 80s	192s ± 75s	0.001
PLR60°	145s ± 58s	212s ± 94s	<0.0001

Data presented as mean ± SD. ANOVA test followed by post hoc Bonferroni test across five positions.

PLR, passive leg raising.

1 Semirecumbent compared with supine (*P* = 0.007).

2 Sitting compared with PLR20° (*P* = 0.037).

3 Paired *t*-test.

## Discussion

Similar to the previous study conducted by Siu et al. (2008), the sitting position was the least appropriate position in which to obtain optimal Doppler signals using USCOM. In the sitting position, not only the aortic SVI was significantly lower than those in the other four positions, but the Fremantle and PWH scores for the aortic measurements were also significantly poorer than those in the other four positions. In the supine position, the heart is displaced cranially and posteriorly, which may facilitate the transducer beam to target the aortic or pulmonary valves and be directed parallel to the flow through the aortic or pulmonary valves. In the sitting and semirecumbent positions, the heart is displaced anteriorly and moves toward the abdomen, leading to difficulty in obtaining optimal alignment between the transducer and the valves, especially the aortic valves. Using the pulmonary approach, the CI in the semirecumbent position was comparable to those in the supine position although the SVI was relatively low which may be due to a shift in blood from the intra- to the extrathoracic compartment (physiological response; Buhre et al. 2000) rather than the improper alignment between the transducer and the valve. Besides, the Fremantle and PWH scores for the pulmonary measurements in the semirecumbent position were similar to those in the supine position. Therefore, it is feasible to use USCOM to measure hemodynamic parameters in the semirecumbent position using the pulmonary approach for subjects with respiratory compromise.

For the pulmonary approach, the transducer should be located at the left parasternal position (between the 2nd to 5th intercostal spaces) with more variation in position than for the aortic approach. Therefore, the time required to obtain three Doppler signals with the highest SVIs using the pulmonary approach was longer than those using the aortic approach in all positions. Nevertheless, the image acquisition is generally well tolerated using the pulmonary approach. Also, some subjects find pressure in the suprasternal notch uncomfortable, particularly those with respiratory distress.

The SVIs in the PLR 20° and PLR 60° positions were similar with those in the supine position. PLR did not cause a statistically significant increase in the SVI regardless of the degree of leg elevation. These findings were inconsistent with previous studies (Boulain et al. 2002; Monnet and Teboul 2008). When the leg elevation is prolonged, the effect of PLR on SVI may not be always sustained (Monnet and Teboul 2008). The hemodynamic effect of PLR has been reported to lessen with time, rarely exceeding 10 min (Boulain et al. 2002). However, sometimes more than 10 min elapsed from the onset of PLR to the completion of the USCOM measurement in this study. Any increase in SVI induced by PLR might be too transient to be detected before obtaining three Doppler signals. Therefore, it was impossible to conclude from this study whether the degree of leg elevation could influence the hemodynamic effect of PLR. In view of the rapid PLR preload effect, the changes in SVI induced by PLR should be continuously monitored after the onset of PLR.

Pathophysiological changes of the heart structures in subjects with cardiac diseases or structural changes related to previous cardiac surgery may complicate the USCOM measurement and mask the potential effects of positioning. Therefore, these subjects were not recruited in this study. As a result, the findings may not be reflective of the entire population. In order to validate the generalization of our results, further studies in larger populations are recommended. The accuracy of the haemodynamic measurements was not compared with any reference method such as pulmonary artery catheter (PAC). However, this may not be realistic as it would be unethical and impractical to subjects with no known acute illness to such an invasive and inherently risky procedure.

## Conclusion

The supine position is the suggested position for measurement of hemodynamic parameters using USCOM. However, for subjects who are unable to maintain the supine position, it is feasible to perform USCOM measurements using the pulmonary approach in the semirecumbent position. The sitting and semirecumbent positions are the least suitable and reliable positions to perform USCOM measurements using the aortic approach. Further studies in larger populations are recommended to validate the generalization of our results.
